# A comparative study of intravenous polymyxin B and colistimethate sodium on efficacy, safety, and cost comparison in treating pulmonary infections: a retrospective cohort study

**DOI:** 10.3389/fphar.2026.1726159

**Published:** 2026-05-13

**Authors:** Chen Wang, Xing Guo, Lin Li, Rui Yang

**Affiliations:** 1 Department of Clinical Pharmacy, The First Affiliated Hospital of Shandong First Medical University and Shandong Provincial Qianfoshan Hospital, Jinan, China; 2 Department of Clinical Pharmacy, The 960th Hospital of the Joint Logistic Support Force of the Chinese People’s Liberation Army, Jinan, China; 3 Department of Pharmacy, The People’s Hospital of Rizhao City, Rizhao, China; 4 Department of Pharmacy, The People’s Hospital of Binzhou City, Binzhou, China

**Keywords:** colistimethate sodium, cost comparison, efficacy, polymyxin B sulfate, safety

## Abstract

Pulmonary infections have become a major global health challenge due to their high incidence, and the escalating bacterial resistance has further increased the difficulty of clinical treatment. As a pivotal agent for treating infections caused by multidrug-resistant Gram-negative bacteria, polymyxins hold a critical position in the management of pulmonary infection. This retrospective cohort study aimed to compare the efficacy, safety, and cost comparison of polymyxin B sulfate (PMB) and colistimethate sodium (CMS) in the treatment of pulmonary infections, providing real-world evidence to support clinical decision-making. A total of 527 inpatients diagnosed with pulmonary infections were included. Univariate and multivariate logistic regression analyses were performed to assess treatment outcomes and identify risk factors associated with all-cause mortality and nephrotoxicity. The results showed that there were no statistically significant differences between the two groups in 28-day and 42-day all-cause mortality or clinical response rate, whereas the PMB group was associated with a significantly shorter length of hospital stay. Univariate and multivariate logistic regression analyses on the factors influencing 28-day all-cause mortality revealed that concomitant extracorporeal membrane oxygenation (ECMO) (OR = 5.312, 95% CI: 1.412–22.893, P = 0.018), hypoalbuminemia (OR = 2.641, 95% CI: 1.549–4.613, P < 0.001), intensive care unit (ICU) admission (OR = 2.579, 95% CI: 1.273–5.330, P = 0.009), central nervous system infection (OR = 8.577, 95% CI: 3.198–24.254, P < 0.001) and concomitant use of immunosuppressants (OR = 4.113, 95% CI: 2.498–6.925, P < 0.001) significantly increased the risk of death in patients. In contrast, adjuvant inhaled polymyxin E therapy and prolonged duration of polymyxin administration exhibited a protective effect. Regarding safety, the incidence of hepatotoxicity was similar between the two groups. Although the difference in nephrotoxicity incidence was not statistically significant, the CMS group showed a numerically higher trend. In addition, the incidence of hematologic toxicity was significantly higher in the CMS group than in the PMB group. Univariate and multivariate logistic regression analyses identified the factors influencing nephrotoxicity, indicating that concomitant dialysis (OR = 2.539, 95% CI: 1.461–4.442, P = 0.001), hypoalbuminemia (OR = 2.620, 95% CI: 1.542–4.596, P = 0.001) and concomitant use of diuretics (OR = 3.022, 95% CI: 1.921–4.837, P < 0.001) were independent risk factors for nephrotoxicity. Regarding economic aspects, following the implementation of the national volume-based procurement (VBP) policy, the procurement cost of PMB was significantly lower than that of CMS (P < 0.05), indicating a clear economic advantage for PMB. No significant difference was observed in total hospitalization costs between the two groups. In conclusion, in the treatment of pulmonary infections in adults, PMB and CMS show comparable overall efficacy, while PMB is associated with a significantly shorter length of hospital stay and presents advantages in safety and cost outcomes. Limited by the retrospective observational design, the above conclusions still require further validation in prospective studies.

## 1 Introduction

Pulmonary infections represent a major global health challenge due to their high incidence, with specific risk factors including immunosuppression, prolonged hospitalization, and mechanical ventilation (Povoa et al., 2024). The growing problem of bacterial resistance has rendered numerous commonly used antibacterial agents less effective, compounding the challenges faced in clinical therapy (Bassetti et al., 2017; Azoulay et al., 2020). The clinical importance of polymyxins, a key class of drugs targeting multidrug-resistant Gram-negative bacteria, is paramount in the management of pulmonary infections, often establishing them as the final line of defense (Nang et al., 2021; Slingerland and Martin, 2024). The polymyxin antibiotics comprise polymyxin B sulfate (PMB), colistimethate sodium (CMS), and polymyxin E sulfate (PME), with PMB and CMS being the predominant agents employed clinically. Research has revealed notable differences between PMB and CMS in their molecular structures, pharmacokinetic profiles, and therapeutic outcomes (Tran et al., 2016; Zhang et al., 2021). As PMB is administered in its active form, it circumvents the need for *in vivo* conversion, thereby achieving rapid bactericidal activity and attaining therapeutic plasma levels quickly (Tran et al., 2016). Consequently, PMB is advantageous for systemic infections and is particularly suited for bloodstream infections; despite this benefit, its use carries a considerable risk of nephrotoxicity and neurotoxicity (Chang et al., 2022; Zhang et al., 2024). CMS is a prodrug that requires conversion to its active form, polymyxin E, *in vivo* to exert its antibacterial effect. This activation process results in pharmacokinetic properties that are highly dependent on renal function (Grégoire et al., 2017). As a prodrug, CMS is primarily converted to its active form, polymyxin E, in the urinary tract, resulting in high bactericidal concentrations that make it particularly effective for treating urinary tract infections. Nevertheless, both polymyxin agents are clinically used for pulmonary infections. Currently, there is a scarcity of robust clinical data directly comparing the efficacy of intravenous polymyxin B sulfate (PMB) and colistimethate sodium (CMS) for this specific indication (Bu et al., 2025). Therefore, this study conducted a retrospective cohort analysis to compare the efficacy and safety of these two agents. The aim was to provide robust evidence to guide the treatment of multidrug-resistant Gram-negative bacterial pulmonary infections, optimize therapeutic strategies, and ultimately improve patient management.

## 2 Materials and methods

### 2.1 Study design and patients

From January 2020 to December 2024, patients with pulmonary infection who received intravenous administration of PMB or CMS at the First Affiliated Hospital of Shandong First Medical University (Shandong Qianfoshan Hospital) were enrolled. The drug regimens for the two groups were as follows:

Polymyxin B Sulfate (Shanghai No. One Biochemical & Pharmaceutical Co., Ltd.), specification: 500,000 units per vial. The original price was 2303 RMB per vial, which was adjusted to 123 RMB per vial in January 2024. The median daily dose administered to patients was 1.44 mg/kg (interquartile range: 1.25–1.71 mg/kg).

Colistimethate Sodium (Zhengda Tianqing Pharmaceutical Group Co., Ltd.), specification: 150 mg per vial. The original price was 1988 RMB per vial, which was adjusted to 1,298.56 RMB per vial in January 2024. The median daily dose administered to patients was 4.02 mg/kg (interquartile range: 3.43–4.54 mg/kg).

Inclusion Criteria: patients were included if they had a diagnosed pulmonary infection and received intravenous therapy with either PMB or CMS for more than 3 days during their hospitalization.

Exclusion Criteria: Patients were excluded for the following reasons: concurrent intravenous use of both polymyxins, incomplete treatment data (including missing laboratory parameters), or if they were pregnant or lactating.

### 2.2 Data extraction

All data were obtained from the hospital’s medical big data cloud platform for single diseases. This platform integrates data from multiple systems, including the Hospital Information System (HIS), Electronic Medical Record (EMR), Laboratory Information System (LIS), Picture Archiving and Communication Systems (PACS), and Nursing Information System (NIS). For this study, data on the baseline characteristics, laboratory test results, and disease treatment progress of the enrolled patients were collected.

Data were independently extracted and cross-validated by two investigators. The extracted variables included demographic characteristics, comorbidities, infection-related indicators, laboratory test results, medication regimens, treatment duration, hospitalization outcomes, and adverse events. Data extraction was performed in strict accordance with the predefined study protocol, and missing data and outliers were managed using a uniform standardized procedure.

This study was approved by the hospital’s ethics committee (YXLLKY-2022–026), and informed consent was waived.

### 2.3 Outcome measures

All-cause mortality was defined as death during hospitalization or withdrawal of treatment due to disease progression or other reasons at 28 days and 42 days after the initiation of PMB or CMS. Clinical response was defined as the return of clinical symptoms and signs to normal or improvement compared with pre-treatment, along with normal or improved results of etiological tests and biochemical tests relative to pre-treatment. The evaluation criteria for safety endpoints were based on the Common Terminology Criteria for Adverse Events (CTCAE) Version 5.0 (Institute, 2017).

### 2.4 Statistical analysis

Statistical analyses in this study were performed using the hospital’s medical big data cloud platform for single diseases (based on R software). Continuous data following a normal distribution were expressed as $\bar{x}$ ±s, and comparisons between groups were conducted using the independent samples t-test. Continuous data not following a normal distribution were presented as median M (P25, P75), with inter-group comparisons performed *via* the Wilcoxon rank-sum test. Categorical data were expressed as counts n (%), and group comparisons were made using the *χ* ^*2*^ test or Fisher’s test. Multivariate logistic regression analysis was used to adjust for baseline variables with statistical differences, eliminating the impact of baseline imbalance on the results. Univariate analysis and multivariate logistic regression analysis were separately conducted for factors influencing all-cause mortality and nephrotoxicity to identify potential risk factors. A two-tailed P-value < 0.05 was considered statistically significant.

## 3 Results

**FIGURE 1:**
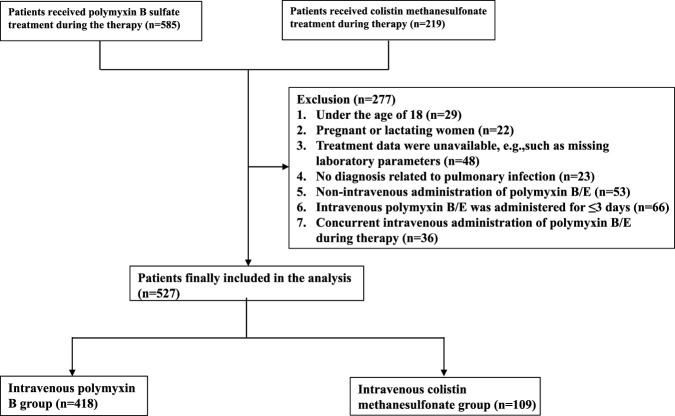
Schematic diagram of the patient screening process.

This study included 527 patients with pulmonary infections who received intravenous PMB or CMS for ≥3 days. The cohort comprised 418 patients in the PMB group and 109 in the CMS group (Figure 1). Baseline characteristics differed significantly between groups, including age and concomitant hypoalbuminemia (P < 0.05). These discrepancies were addressed in the outcome analysis using univariate and multivariate logistic regression. After adjustment, baseline characteristics were well-balanced (Table 1).

**TABLE 1:** Baseline characteristics of patients in the PMB and CMS groups.

Characteristics	PMB group (n = 418)	CMS group (n = 109)	Total (n = 527)	*P value*
Demographic features				
Age (years), M (P25, P75)	66 (53.78)	63 (49,72)	65 (53.77)	0.024
Male (%)	303 (72.5)	73 (67.0)	376 (71.3)	0.257
Comorbidities (%)				
Chronic lung disease	48 (11.5)	24 (22.0)	72 (13.7)	0.022
Asthma	7 (1.7)	1 (0.9)	8 (1.5)	0.892
Chronic liver disease	96 (23.0)	30 (27.5)	126 (23.9)	0.321
Chronic kidney disease	129 (30.9)	25 (22.9)	154 (29.2)	0.105
Diabetes	146 (34.9)	20 (18.3)	166 (31.5)	<0.01
Hematological malignancy	9 (2.2)	13 (11.9)	22 (4.2)	<0.001
Concomitant conditions (%)				
Transplant status	25 (6.0)	8 (7.3)	33 (6.3)	0.602
Extracorporeal membrane oxygenation (ECMO)	15 (3.6)	3 (2.8)	18 (3.4)	0.895
Mechanical ventilation	301 (72.0)	55 (50.5)	356 (67.6)	<0.01
Shock	131 (31.3)	21 (19.3)	152 (28.8)	0.013
ICU admission	288 (68.9)	53 (48.6)	341 (64.7)	<0.01
Hypoalbuminemia	267 (63.9)	90 (82.6)	357 (67.7)	<0.01
Pathogens (%)				
*Escherichia coli*	162 (38.8)	42 (38.5)	204 (38.7)	0.966
*Klebsiella pneumoniae*	220 (52.6)	49 (45.0)	269 (51.0)	0.153
*Enterobacter cloacae*	17 (4.1)	1 (0.9)	18 (3.4)	0.107
*Pseudomonas aeruginosa*	185 (44.3)	44 (40.4)	229 (43.5)	0.465
*Acinetobacter* baumannii	255 (61.0)	40 (36.7)	295 (56.0)	<0.01
Concomitant infection sites (%)				
Bloodstream infection	135 (32.3)	26 (23.9)	161 (30.6)	0.088
Intra-abdominal infection	56 (13.4)	4 (3.7)	60 (11.4)	0.004
Urinary tract infection	34 (8.1)	5 (4.6)	39 (7.4)	0.208
Central nervous system infection	28 (6.7)	3 (2.8)	31 (5.9)	0.119
Combination medications (%)				
Meropenem	49 (11.7)	17 (15.6)	66 (12.5)	0.276
Ceftazidime-avibactam	12 (2.9)	5 (4.6)	17 (3.2)	0.366
Tigecycline	56 (13.4)	18 (16.5)	74 (14)	0.404
Inhalation CMS	22 (5.3)	35 (32.1)	57 (10.8)	<0.01
Inhalation PMB	37 (8.9)	—	37 (7.0)	0.001
Cefoperazone-sulbactam	83 (19.9)	30 (27.5)	113 (21.4)	0.082
Piperacillin-tazobactam	42 (10.0)	12 (11.0)	54 (10.2)	0.768
Immunosuppressants	163 (39.0)	68 (62.4)	231 (43.8)	<0.01
Other indicators				
PMB/CMS treatment duration (days), M (P25, P75)	8 (5,11)	7 (5,9)	7 (5,10)	0.363

Data are presented as n (%) for categorical variables and median (25th percentile, 75th percentile) for continuous variables.

P *values* were calculated using the chi-square test for categorical variables and the Wilcoxon rank-sum test for continuous variables.

Abbreviations: PMB, Polymyxin B sulfate; CMS, colistimethate sodium; ECMO, extracorporeal membrane oxygenation; ICU, intensive care unit; M, median; P25, 25th percentile; P75, 75th percentile.

**FIGURE 2:**
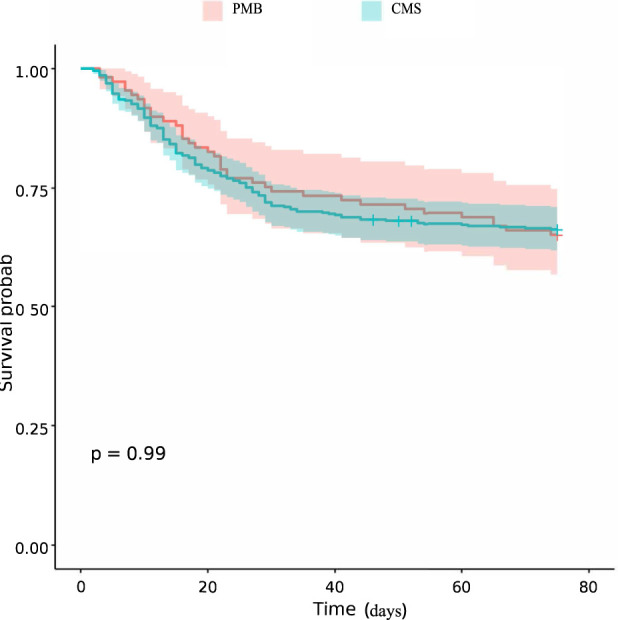
Kaplan-Meier survival curves of patients receiving intravenous PMB or CMS.

The primary outcomes were 28- and 42-day all-cause mortality, clinical response rate, and hospital length of stay (LOS). Before adjustment, the median LOS was significantly shorter in the PMB group than in the CMS group (16 days vs. 25 days; P < 0.001). After adjusting for baseline confounding factors using multivariate regression analysis, the between-group difference in LOS remained statistically significant, indicating that PMB had a significant advantage in shortening hospital stay. Furthermore, no significant intergroup differences were observed in clinical response rate (42.6% vs. 47.7%; P = 0.645), 28-day mortality (32.8% vs. 31.2%; P = 0.853), or 42-day mortality (33.7% vs. 34.9%; P = 0.851) (Table 2). Consistently, Kaplan–Meier survival analysis showed no significant difference in survival time between the two groups (Figure 2).

**TABLE 2:** Comparison of efficacy outcomes between PMB and CMS groups.

Efficacy (%)	PMB group (n = 418)	CMS group (n = 109)	Total (n = 527)	*P value*	Multivariate logistic regression		
					OR	95% CI	*P value*
28-day all-cause mortality	137 (32.8)	34 (31.2)	171 (32.4)	0.753	1.065	0.550–2.086	0.853
42-day all-cause mortality	141 (33.7)	38 (34.9)	179 (34.0)	0.824	0.939	0.487–1.815	0.851
Clinical response rate	178 (42.6)	52 (47.7)	230 (43.6)	0.337	1.137	0.659–1.971	0.645

Efficacy (%)	PMB group (n = 418)	CMS group (n = 109)	Total (n = 527)	*P value*	Multivariate logistic regression		
					*β*	Standard deviation	*P value*
Length of hospital stay (days), M (P25, P75)	16 (10,28)	25 (16,35)	18 (11,29)	<0.001	−4.459	1.611	0.006

Continuous data are presented as median (P25, P75), and categorical data as n (%).

P *values* were calculated using the χ^2^ test for categorical variables and the Mann–Whitney U test for continuous variables. Multivariate logistic regression was used for binary outcomes (mortality, clinical response), and linear regression for length of hospital stay.

Abbreviations: OR, odds ratio; CI, confidence interval; *β*, regression coefficient; SD, standard deviation.

Univariate and multivariate logistic regression analyses identified several factors independently associated with 28-day all-cause mortality (Table 3). The combination with ECMO (OR = 5.312, 95% CI: 1.412–22.893, P = 0.018), hypoalbuminemia (OR = 2.641, 95% CI: 1.549–4.613, P < 0.001), ICU admission (OR = 2.579, 95% CI: 1.273–5.330, P = 0.009), CNS infection (OR = 8.577, 95% CI: 3.198–24.254, P < 0.001), and concurrent use of immunosuppressants (OR = 4.113, 95% CI: 2.498–6.925, P < 0.001) were independent risk factors for mortality. In contrast, adjuvant inhaled polymyxin E therapy (OR = 0.247, 95% CI: 0.099–0.562, P = 0.001) and a longer duration of polymyxin treatment (OR = 0.848, 95% CI: 0.797–0.898, P < 0.001) emerged as protective factors.

**TABLE 3:** Univariate and multivariate analyses of factors associated with 28-day all-cause mortality.

Influencing factor	Univariate analysis		Multivariate logistic regression		
	χ2/w value	*P* value	OR	95% CI	*P* value
Chronic kidney disease	16.79	<0.001	1.520	0.933–2.476	0.092
ECMO	6.98	0.008	5.312	1.412–22.893	0.018
Mechanical ventilation	23.68	<0.001	1.487	0.728–3.067	0.278
Shock	21.70	<0.001	1.424	0.847–2.396	0.182
ICU admission	24.36	<0.001	2.579	1.273–5.330	0.009
Hypoalbuminemia	27.11	<0.001	2.641	1.549–4.613	<0.001
*Pseudomonas aeruginosa*	7.21	0.007	0.793	0.499–1.255	0.323
Bloodstream infection	6.64	0.010	1.113	0.667–1.844	0.680
Central nervous system infection	7.53	0.006	8.577	3.198–24.254	<0.001
Tigecycline	4.58	0.032	1.321	0.698–2.475	0.387
Inhalation CMS	5.04	0.025	0.247	0.099–0.562	0.001
Piperacillin-tazobactam	5.32	0.021	0.547	0.225–1.228	0.161
Immunosuppressants	14.13	<0.001	4.113	2.498–6.925	<0.001
PMB/CMS treatment duration (days)	40,636.50	<0.001	0.848	0.797–0.898	<0.001

The χ^2^/w values correspond to the test statistic from the chi-square test or Wilcoxon rank-sum test in univariate analysis. *P* values in the univariate analysis represent between-group comparisons. *P* values in the multivariate logistic regression represent the significance of each variable after adjustment for confounders.

Secondary endpoints were safety—assessed by the incidence of nephrotoxicity, hepatotoxicity, and hematotoxicity—and cost comparison, encompassing polymyxin drug costs and total hospitalization expenses. A significantly higher incidence of hematotoxicity, primarily thrombocytopenia, was observed in the CMS group compared to the PMB group (35.8% vs. 22.2%; P = 0.018). Although the differences did not reach statistical significance, nephrotoxicity and hepatotoxicity incidences were numerically higher in the CMS group, with nephrotoxicity being conspicuously more prominent (Table 4).

**TABLE 4:** Comparison of safety outcomes between the PMB and CMS groups.

Safety (%)	PMB group (n = 418)	CMS group (n = 109)	Total (n = 527)	*P value*	Multivariate logistic regression		
					OR	95% CI	*P value*
Nephrotoxicity							
Total nephrotoxicity	109 (26.1)	34 (31.2)	143 (27.1)	0.285	0.604	0.317–1.144	0.122
Acute kidney injury	69 (16.5)	25 (22.9)	94 (17.8)	0.118	0.628	0.302–1.310	0.212
Elevated serum creatinine	55 (13.2)	21 (19.3)	76 (14.4)	0.106	0.541	0.247–1.197	0.125
Elevated blood urea	74 (17.7)	27 (24.8)	101 (19.2)	0.095	0.553	0.273–1.122	0.099
Hepatotoxicity							
Total hepatotoxicity	95 (22.7)	23 (21.1)	118 (22.4)	0.717	1.343	0.663–2.790	0.42
Elevated ALT	30 (7.2)	11 (10.1)	41 (7.8)	0.312	1.218	0.415–3.889	0.728
Elevated AST	30 (7.2)	5 (4.6)	35 (6.6)	0.333	2.195	0.670–8.500	0.219
Elevated total bilirubin	40 (9.6)	9 (8.3)	49 (9.3)	0.674	1.041	0.374–3.076	0.94
Elevated γ-glutamyl transferase (GGT)	48 (11.5)	17 (15.6)	65 (12.3)	0.245	0.623	0.281–1.414	0.248
Hematotoxicity							
Total hematotoxicity	93 (22.2)	39 (35.8)	132 (25.0)	0.004	0.471	0.251–0.881	0.018
Decreased hemoglobin (HGB)	33 (7.9)	19 (17.4)	52 (9.9)	0.003	0.353	0.153–0.819	0.014
Decreased platelet count (PLT)	71 (17.0)	24 (22.0)	95 (18.0)	0.223	0.748	0.368–1.545	0.425

ALT, alanine aminotransferase; AST, aspartate aminotransferase. Statistical methods and null hypotheses for P values are the same as those in Table 2.

Univariate and multivariate logistic regression analyses identified several independent risk factors for nephrotoxicity (Table 5). These included concurrent dialysis (OR = 2.539, 95% CI: 1.461–4.442, *P* = 0.001), hypoalbuminemia (OR = 2.620, 95% CI: 1.542–4.596, *P* = 0.001), and concomitant diuretic use (OR = 3.022, 95% CI: 1.921–4.837, *P* < 0.001).

**TABLE 5:** Univariate and multivariate logistic regression analyses of factors associated with nephrotoxicity.

Influencing factor	Univariate analysis		Multivariate logistic regression		
	χ2/w value	*P* value	OR	95% CI	*P* value
Age (years)	—	0.013	1.001	0.987–1.015	0.930
Chronic liver disease	13.94	<0.010	1.370	0.820–2.274	0.226
Chronic kidney disease	29.99	<0.010	1.263	0.747–2.116	0.379
Coronary heart disease	4.28	0.039	0.895	0.543–1.466	0.662
Dialysis state	42.59	<0.010	2.539	1.461–4.442	0.001
ECMO	4.86	0.028	1.088	0.383–3.054	0.872
ICU admission	22.86	<0.010	1.688	0.972–2.973	0.066
Hypoalbuminemia	28.50	<0.010	2.620	1.542–4.596	0.001
*Escherichia coli*	18.61	<0.010	1.466	0.943–2.278	0.089
*Klebsiella pneumoniae*	7.40	0.007	1.330	0.862–2.056	0.198
*Acinetobacter* baumannii	4.63	0.032	0.880	0.553–1.394	0.585
Novel coronavirus	4.20	0.041	1.143	0.570–2.252	0.703
Bloodstream infection	18.59	<0.010	1.505	0.956–2.358	0.076
Proton pump inhibitor	10.47	0.001	1.310	0.802–2.169	0.287
Diuretic	29.54	<0.010	3.022	1.921–4.837	<0.001

χ^2^/w represents the test statistic from the chi-square test or Wilcoxon rank-sum test in univariate analysis. *P* values and statistical methods are the same as those in Table 3.

Given comparable efficacy between the two groups, cost-minimization analysis (CMA) was performed after adjusting for baseline confounding factors using multivariate logistic regression to evaluate their economic outcomes (Table 6).

**TABLE 6:** Economic outcomes between PMB and CMS groups before and after price adjustment.

Economic (before price adjustment)	PMB group (n = 378)	CMS group (n = 83)	*P value*	Multivariate logistic regression		
				*β*	Standard error	*P value*
PMB/CMS cost	32,242 (18424,46,060)	18,179.84 (11687,28,568)	<0.001	−12048	2,461	<0.001
Total hospitalization cost	149,234 (106,788,234,921)	164,924 (92049,269,766)	0.991	5,813	16,712	0.728

Economic (after price adjustment)	PMB group (n = 40)	CMS group (n = 26)	*P value*	Multivariate logistic regression		
				*β*	Standard error	*P value*
PMB/CMS cost	2,583 (1599,3571)	19,478 (10695,25,971)	<0.001	12,558	2,553	<0.001
Total hospitalization cost	138,739 (86247,246,004)	147,451 (94272,209,624)	0.922	14,403	50,556	0.778

Data are presented as median (P25, P75). Group comparisons were performed using the Mann–Whitney U test Multivariate linear regression was used to analyze cost predictors.

*β*, regression coefficient; SE, standard error. Costs are expressed in Chinese Yuan (CNY).

Before the 2024 price adjustment of PMB.

1. Polymyxin cost: The median cost in the PMB group was significantly higher than that in the CMS group (P < 0.001), indicating a lower procurement cost for CMS.
2. Total hospitalization cost: The median total hospitalization cost in the PMB group was lower than that in the CMS group, with no statistically significant difference (P = 0.345).

After the 2024 price adjustment of PMB.

1. Polymyxin cost: The median cost in the PMB group was significantly lower than that in the CMS group (P < 0.001), suggesting a marked reduction in the procurement cost of PMB after January 2024.
2. Total hospitalization cost: The median total hospitalization cost in the PMB group was also lower than that in the CMS group, with no statistically significant difference (P = 0.591).

Overall, after the 2024 price adjustment of PMB, both the total hospitalization cost and polymyxin cost in the PMB group were lower than those in the CMS group, suggesting that PMB is more economical from a healthcare perspective.

## 4 Discussion

The misuse of antimicrobial agents has propelled the relentless emergence of drug-resistant bacteria, particularly multidrug-resistant (MDR) and extensively drug-resistant (XDR) strains. This evolving crisis has profoundly complicated clinical management and poses a grave threat to global public health (Theuretzbacher, 2025). Carbapenems were historically considered the last-line defense against Gram-negative bacilli. However, the rapid emergence of carbapenem-resistant strains has led to a critically dwindling arsenal of effective therapeutic agents (Mills and Marchaim, 2021). Pulmonary infections caused by multidrug-resistant Gram-negative bacilli pose a significant threat due to their high transmissibility, severity, and therapeutic complexity, often leading to poor patient outcomes. Polymyxins have re-emerged as a critical therapeutic option for these infections, owing to their favorable resistance profile and potent bactericidal activity (Zhou et al., 2024), but their clinical application is often limited by nephrotoxicity (Asdahl et al., 2020).

A systematic meta-analysis pooled data from nine clinical studies (comprising 672 patients). It demonstrated that therapy with IV combined with nebulized polymyxin was associated with significantly higher clinical response and pathogen eradication rates, as well as lower all-cause mortality, compared to IV monotherapy (Liu et al., 2015). While both our study and that of Wei Bu et al. (Bu et al., 2025) evaluated polymyxins in ICU patients with CRAB infections, a key methodological difference lies in their use of PSM to balance baseline characteristics. Notably, their findings corroborate the significance of treatment duration, demonstrating that a polymyxin course of ≤7 days independently predicts higher 28-day mortality, thereby affirming its role as a pivotal factor in managing these severe infections. Analysis of data from 527 patients revealed no statistically significant differences in efficacy endpoints between the PMB and CMS groups (P > 0.05). However, further investigation identified that combined nebulized colistin and a longer duration of intravenous polymyxin therapy were independently associated with reduced in-hospital mortality. These findings suggest that an optimized regimen combining nebulized colistin with an adequate course of intravenous therapy may improve patient prognosis. Consequently, enhanced monitoring and tailored treatment protocols should be considered for high-risk patients.

Furthermore, our study identified several independent risk factors for increased mortality, including concurrent central nervous system (CNS) infection, dialysis, ECMO support, ICU admission, hypoalbuminemia, and concomitant immunosuppressant use. ECMO support and ICU admission reflect greater disease severity and were associated with higher mortality. Notably, concurrent CNS infection conferred the greatest risk (OR = 8.857), indicating a nearly ninefold increase in mortality and underscoring the critical need for vigilant monitoring and aggressive management in these patients. This is particularly relevant for bacterial meningitis, a common CNS infection with distinct epidemiological patterns (Goodlet and Nailor, 2024). Previous studies have identified advanced age, hypotension, seizures, and the absence of antimicrobial therapy prior to admission as factors associated with increased mortality in patients with bacterial meningitis (Hasbun, 2022). Our results emphasize the critical need for personalized treatment regimens in managing CNS infections, given their unique severity. This is further supported by the role of hypoalbuminemia, an independent risk factor we identified, which is a well-established predictor of poor outcomes in severe acute conditions like septic shock, heart failure, and acute coronary syndromes (Mirzai et al., 2024). Given its higher protein binding affinity, polymyxin B experiences a disproportionate rise in free drug concentration during hypoalbuminemia compared to polymyxin E, indicating its pharmacokinetics are more significantly altered by this condition (Pi et al., 2023). The multifactorial nature of hypoalbuminemia’s impact on prognosis encompasses inflammatory, nutritional, hepatic, hemodynamic, immunological, and cardiac pathways. The interplay among these mechanisms potentiates their individual effects, thereby accelerating clinical decline and increasing the propensity for fatal outcomes (Ostermann et al., 2019).

In terms of safety, we found that the risk of hematological toxicity in the polymyxin E group was significantly higher than that in the polymyxin B group (P < 0.05). Although hematological system-related toxicity is not mentioned in the package inserts of either drug, through a review of relevant literature, we noted that Serhanhad reported a case of acute thrombocytopenia in a 5-year-old girl during treatment with polymyxin E. This finding reminds us that polymyxin E may be associated with thrombocytopenia (Kupeli, 2015); therefore, when using this drug for treatment, clinicians should maintain a high level of vigilance and closely monitor patients’ platelet counts to ensure patient safety. In addition, regarding nephrotoxicity, although no statistically significant difference was shown between the two groups after multivariate analysis, the CMS group exhibited a higher trend of nephrotoxicity than the PMB group in terms of factors related to AKI (P = 0.065). Our study further revealed that concurrent dialysis, hypoalbuminemia, and the use of diuretics are all independent risk factors for increased nephrotoxicity. Previous studies have shown that polymyxins can induce nephrotoxicity by mechanisms such as disrupting the structure of renal tubular cell membranes and inducing cell apoptosis, with the incidence of nephrotoxicity ranging from 20% to 60% (Sisay et al., 2021; Ballı et al., 2024). Polymyxin B is mainly metabolized *via* non-renal pathways in the body, with only 4% excreted through the kidneys. Therefore, the metabolism of polymyxin B may be less affected by renal function. In a prospective study conducted, it was found that the incidence of nephrotoxicity in the polymyxin E treatment group was 39.3%, while that in the polymyxin B group was only 11.8% (Aggarwal and Dewan, 2018). There was little difference between the two groups in terms of the time to onset of nephrotoxicity; further dose-response analysis showed that when the daily dose of polymyxin E was ≥300 mg, the risk of nephrotoxicity increased significantly and showed a dose-dependent relationship. In our study, the incidence of nephrotoxicity was 31.2% in the CMS group, compared with 26.1% in the PMB group. This result further indicates that polymyxin E has a higher nephrotoxic potential than polymyxin B, providing a reference for clinical drug selection. Hypoalbuminemia may lead to an increase in drug volume of distribution and clearance, resulting in elevated free drug concentrations, which in turn indirectly increases the risk of nephrotoxicity (Lionetti et al., 2020; Deniz and Alişik, 2024). This study also found that the proportion of patients with AKI was significantly higher in those using diuretics; the possible mechanisms involved include hemodynamic effects, electrolyte disturbances, and drug-drug interactions (Deniz and Alişik, 2024). Thus, in clinical practice, these factors should be comprehensively considered, and corresponding preventive and interventional measures should be implemented to reduce the risk of related kidney injury, protect patients’ health, and improve their prognosis.

From the perspective of the healthcare system, direct medical costs were adopted in this study, including polymyxin costs and total hospitalization costs. Given that there was no statistically significant difference in efficacy between the PMB and CMS groups, cost-minimization analysis (CMA) was used for the economic evaluation. During hospitalization, there was no significant difference in total hospitalization costs between the two groups. However, after price adjustment under the national volume-based procurement policy, the total cost of PMB was considerably lower than that of CMS, which may be related to the higher unit price of CMS.

In conclusion, this study demonstrates that polymyxin B (PMB) and colistimethate sodium (CMS) have comparable clinical efficacy in the treatment of pulmonary infections. Notably, the length of hospital stay was significantly shorter in the PMB group than in the CMS group. Moreover, PMB shows superior advantages in safety and economics: it is associated with a lower incidence of hematological toxicity and presents a more distinct cost advantage under the national volume-based procurement policy, providing a reasonable reference for clinical drug selection. Notably, this study is a retrospective observational study with potential residual confounding factors. Therefore, the aforementioned advantages of PMB should be comprehensively evaluated in combination with clinical practice and cannot be used as the sole basis for drug selection. Further multicenter, prospective, large-sample studies are warranted to verify these findings, explore optimal dosage and administration strategies, refine individualized treatment regimens, and improve clinical outcomes in patients with multidrug-resistant infections.

However, this study has certain limitations. First, this study is a single-center retrospective cohort study. Although multivariate regression analysis was used to adjust for baseline imbalances, selection bias and residual confounding factors cannot be completely avoided, which may affect the objectivity and generalizability of the research conclusions. Second, due to the inherent limitations of the retrospective study design, some key variables (such as the specific operation details of nebulized medication and long-term follow-up data) were not fully recorded, which may have an impact on the accuracy of the analysis results. Third, the economic analysis only included drug procurement costs and short-term hospitalization expenses, without a comprehensive assessment of long-term economic outcomes and indirect medical costs; in addition, the generalizability of our economic findings is limited by regional pricing policies, which may lead to an incomplete evaluation of the relative economic advantages. Based on the above limitations, future studies should adopt a multi-center, prospective research design, supplement long-term follow-up data, and further improve the comprehensiveness and reliability of the research results.

## Data Availability

The datasets generated and analyzed in this study are not publicly available due to patient privacy and data protection regulations. Anonymized, de-identified data may be made available from the corresponding author upon reasonable request and with approval from the institutional review board.
